# Clinical and echocardiographic phenotype of cardiac wasting in patients with advanced cancer

**DOI:** 10.1002/ejhf.3744

**Published:** 2025-08-06

**Authors:** Markus S. Anker, Muhammad Shahzeb Khan, Anja Nikolski, Jan Porthun, Muhammad Sameer Arshad, Sara Hadzibegovic, Alessia Lena, Lucie Kretzler, Jonathan L. Hella, Marco Witkowski, Kathrin Rieger, Johann Ahn, Dominik P. Modest, Ulrich Keller, Lars Bullinger, Matthias Totzeck, Amir A. Mahabadi, Tienush Rassaf, Nikolaus Buchmann, Philipp Attanasio, Tanja Zeller, Mahir Karakas, Carlo G. Tocchetti, Ursula Wilkenshoff, John G.F. Cleland, Stephan von Haehling, Javed Butler, Ulf Landmesser

**Affiliations:** ^1^ Charité – University Medicine Berlin corporate member of Free University Berlin and Humboldt‐University Berlin Berlin Germany; ^2^ German Centre for Cardiovascular Research partner site Berlin, and Berlin Institute of Health Center for Regenerative Therapies Berlin Germany; ^3^ Department of Cardiology, Angiology and Intensive Care Medicine CBF, German Heart Center Charité Berlin Germany; ^4^ School of Cardiovascular and Metabolic Health University of Glasgow Glasgow UK; ^5^ Baylor Scott and White Heart Hospital Plano Texas USA; ^6^ Baylor College of Medicine Temple Texas USA; ^7^ Baylor Scott and White Research Institute Baylor Scott and White Health Dallas Texas USA; ^8^ Department of Cardiology, Angiology and Intensive Care Medicine Campus Virchow Clinic, German Heart Center Charité Berlin Germany; ^9^ Norwegian University of Science and Technology Gjøvik Norway; ^10^ Dow University of Health Sciences Karachi Pakistan; ^11^ Friede Springer Cardiovascular Prevention Center at Charité Charité University Medicine Berlin Berlin Germany; ^12^ Department of Hematology, Oncology and Cancer Immunology, Charité – University Medicine Berlin corporate member of Free University Berlin and Humboldt University Berlin Berlin Germany; ^13^ German Cancer Consortium (DKTK), partner site Berlin, and German Cancer Research Center (DKFZ) Heidelberg Germany; ^14^ Max Delbrück Center Berlin Germany; ^15^ National Center for Tumor Diseases (NCT), partner site Berlin, and German Cancer Research Center (DKFZ) Heidelberg Germany; ^16^ Department of Cardiology and Vascular Medicine, West German Heart and Vascular Center University Hospital Essen Essen Germany; ^17^ German Center for Cardiovascular Research (DZHK e.V.) partner site Hamburg/Lübeck/Kiel Hamburg Germany; ^18^ University Heart Center Hamburg, Clinic of General and Interventional Cardiology Hamburg Germany; ^19^ Department of Intensive Care Medicine University Medical Center Hamburg‐Eppendorf Hamburg Germany; ^20^ Cardio‐Pulmonary Onco‐Immunology, Department of Internal Medicine and Clinical Complexity, Department of Translational Medical Sciences (DISMET) Federico II University Naples Italy; ^21^ Center for Basic and Clinical Immunology Research (CISI), Interdepartmental Center of Clinical and Translational Sciences (CIRCET), Interdepartmental Hypertension Research Center (CIRIAPA) Federico II University Naples Italy; ^22^ Berlin Institute of Health, Charité – University Medicine Berlin Berlin Germany; ^23^ School of Cardiovascular and Metabolic Health, University of Glasgow Glasgow UK; ^24^ Department of Cardiology and Pneumology University of Göttingen Medical Center Göttingen Germany; ^25^ German Centre for Cardiovascular Research (DZHK) partner site Lower Saxony Germany; ^26^ Baylor Scott and White Research Institute Dallas TX USA; ^27^ University of Mississippi, Jackson University of Mississippi Jackson MS USA

**Keywords:** Cancer, Cardiac wasting cardiomyopathy, Left ventricular mass, Echocardiography, Pathophysiology

## Abstract

**Aims:**

Cardiac wasting‐associated cardiomyopathy in patients with advanced cancer is characterized by loss of left ventricular (LV) mass and independently associated with poor prognosis. Better understanding of this very prevalent cardiomyopathy is urgently needed.

**Methods and results:**

Overall, 398 patients with active, mostly advanced cancer without significant cardiovascular disease (mean LV ejection fraction [LVEF] 64.3 ± 0.2%) or active infection were prospectively examined (mean age 60 ± 1 years, 50% women, body mass index 25.0 ± 0.2 kg/m^2^, 26% cachectic). Patients were categorized and compared by quartiles of LV mass/height^2^. LVEF, global longitudinal strain (GLS), and anticancer therapy naive status were similar across quartiles. Patients in Q1 (lowest LV mass quartile) were younger, more likely cachectic, had lower: BMI, 10‐step stair‐climbing power, tricuspid annular plane systolic excursion (TAPSE), stroke volume, cardiac output, and higher heart rate. In repeat follow‐up assessments after 140 ± 8 days (*n* = 143), LVEF, TAPSE, LV mass, left atrial volume, and GLS were found reduced (all *p* ≤ 0.002). Only in those with above‐median LV mass at baseline, cardiac output and heart rate increased during follow‐up – in those with below‐median LV mass, mitral E/A decreased.

**Conclusions:**

Patients with advanced cancer with low LV mass have a distinct phenotype characterized by lower cardiac chamber volumes, stroke volume, and cardiac output, but normal LVEF and GLS that may be the distinct feature of cardiac wasting‐associated cardiomyopathy.

## Introduction

Cardiac wasting‐associated cardiomyopathy, characterized by the loss of left ventricular (LV) mass, is an emerging concern in patients with advanced stage cancer.^1^, ^2^ The presence of cancer‐related clinical wasting can result in a diverse range of structural and haemodynamic alterations in the heart, which can lead to arrhythmias and heart failure.^3^ Cardiac wasting occurs in ~50% of patients with advanced cancer and is associated with thinning of the LV walls, reduction of LV size, and systemic inflammation.^4^ Patients with cardiac wasting‐associated cardiomyopathy have reduced stroke volume, elevated heart rates, decreased blood pressure, and a higher incidence of anaemia.^5^ These alterations in the body's functions contribute to reduced functional capacity, lower overall quality of life and increased mortality.^2^

The complex relationship between cardiac performance and systemic wasting presents a challenge to the understanding of cardiac wasting‐associated cardiomyopathy in cancer patients. Previous studies have underscored the multifaceted nature of cardiac wasting‐associated cardiomyopathy and its impact on patient outcomes.^6^ Patients with cardiac wasting show increased levels of cytokines including interleukin‐6 and C‐reactive protein.^6^, ^7^ These neurohormonal variations highlight the systemic impact of cardiac wasting and the need of understanding how neurohormonal dynamics affect LV mass loss. Studies have demonstrated that the loss of LV mass, a pivotal component of this cardiomyopathy, may not manifest as overt LV systolic dysfunction.^8^, ^9^ These findings warrant a thorough investigation into the relationship between the loss of LV mass and various clinical and echocardiographic measurements of cardiac wasting‐related cardiomyopathy.

A comprehensive understanding of the clinical features of cardiac wasting‐associated cardiomyopathy could improve understanding the pathophysiology of the disease process and help develop targeted therapies with the aim to improve patient outcomes, both cardiac and overall. Accordingly, we investigated the clinical, laboratory and echocardiographic phenotype linked with cardiac wasting‐associated cardiomyopathy in a substantial cohort of cancer patients, with repeat measurements in a subset.

## Methods

### Patient population

This prospective study included 398 patients with cancer hospitalized in the oncology wards of the Charité Campus Benjamin Franklin/Virchow Klinikum, between September 2017 and September 2023. All patients provided written informed consent for participation in the study. The inclusion criteria were as follows: (1) age ≥18 years, (2) diagnosed with histologically confirmed cancer (no second active cancer within last 5 years), (3) no infection requiring antibiotic treatment, (4) LV ejection fraction (LVEF) ≥50%, (5) no history of cardiovascular disease/events (such as coronary artery disease, severe valve dysfunction, myocardial infarction, heart failure). Patients were excluded if they had chronic obstructive pulmonary disease Gold stage ≥3 except for lung cancer patients. Patients with type 2 diabetes mellitus and controlled hypertension (defined as <160/100 mmHg) were not excluded.

### Study design

All patients were evaluated prospectively and underwent the following: a comprehensive medical history, a physical examination which included measuring body weight, height, and body surface area (BSA), performance status evaluation according to the Eastern Cooperative Oncology Group (ECOG)^10^ and Karnofsky index,^11^ biomarker analysis of blood samples, and an electrocardiographic examination using resting 12‐lead electrocardiogram. Most of the patients that were evaluated had advanced stages of cancer. Union for International Cancer Control (UICC) stages III and IV, Ann Arbor classification stages III and IV, and Durie–Salmon classification stage III were used to define advanced stage cancer. All study participants were offered the opportunity to undergo a follow‐up examination, with the ideal time being 3–6 months (with a maximum of 12 months) since the baseline assessment. Using sex‐specific cut‐offs, all patients were categorized into quartiles according to LV mass adjusted for height squared (height^2^). Cachexia was diagnosed when weight loss was ≥5% in the previous 12 months, as reported by the patients and body mass index (BMI) was <24.0 kg/m^2^ at baseline.^12^, ^13^ This study research was approved by the Charité Ethics Committee and carried out in adherence to the principles outlined in the Declaration of Helsinki.

### Echocardiographic examination and left ventricular mass

Three experienced and well‐trained echocardiographers performed a comprehensive two‐dimensional transthoracic echocardiographic examination using standardized operating procedures. The validity of each LV mass analysis was confirmed by two highly qualified and impartial echo‐sonographers. For echocardiograms, a vivid E95 scanner (GE Healthcare) and a Tomtec system were used. In order to determine the LV mass, the Devereux formula^14^ was used, utilizing the linear measurements of LV end‐diastolic wall thickness and diameter extracted from two‐dimensional parasternal long‐axis views. LV mass is presented in absolute, height^2^‐, and BSA‐adjusted form. Cardiac chamber quantification was performed according to the recommendations of the American Society of Echocardiography and the European Association of Cardiovascular Imaging.^15^

### Follow‐up echocardiographic examination

A subset of the study cohort (*n* = 143) underwent a follow‐up echocardiographic assessment to investigate longitudinal changes in cardiac parameters. To assess changes in LV mass over time, patients were stratified into two groups based on their LV mass at baseline: those below the median LV mass and those above the median LV mass.

### Statistical analyses

The normal distribution of the variables was checked using the Kolmogorov–Smirnov test. One‐way analysis of variance (ANOVA) and Fisher's post‐hoc tests were utilized to evaluate differences between groups. Mean ± standard error of the mean (SEM) for values were presented for variables that were normally distributed. Mann–Whitney U test and the Kruskal–Wallis test were used for variables that were not normally distributed. For non‐normally distributed variables, the data were presented as the median and interquartile range. To compare frequencies, the chi‐square test was employed. Parametric *t*‐tests were employed to analyse changes in LV mass development and other relevant echocardiographic parameters within and between the two groups. Adjustments for height and BSA were made to ensure the robustness of the findings. For all analyses, a *p*‐value of <0.05 was deemed statistically significant. The SAS/STAT software version 9.4 (SAS Institute, Inc., Cary, NC, USA), Stata (StataCorp. 2021, Release 17, College Station, TX, USA), and SPSS software version 26.0 (IBM Corp., Armonk, NY, USA) were used to generate the analyses.

## Results

### Baseline characteristics

This study included 398 patients (50% women, mostly advanced stage III/IV, 82%) with a mean age of 60.2 ± 0.7 years (SEM), and a mean BMI of 25.0 ± 0.2 kg/m^2^ (SEM). One quarter of all patients (*n* = 101; 26%) were cachectic. The majority of patients had an advanced cancer stage (*n* = 325; 82%). Observed frequencies of cancer entities in our study cohort are displayed in online supplementary *Table* *S1*. The primary reasons for hospital admission were administration of anticancer treatment (51%), staging and other diagnostics (30%) and general worsening of patients' clinical condition (19%). A total of 211 (53%) patients underwent baseline examination within the first year after initial cancer diagnosis – mean time since first cancer diagnosis was 33 ± 2.6 months (SEM). Patients were grouped by LV mass/height^2^ quartiles (i.e. into four groups), as assessed by transthoracic echocardiography (quartiles for females: <46.58 g/m^2^, 46.58–<55.34 g/m^2^, 55.34 g/m^2^–<62.88 g/m^2^, ≥61.15 g/m^2^; quartiles for males: <56.59 g/m^2^, 56.59–<65.7 g/m^2^, 65.7 g/m^2^–77.15 g/m^2^, ≥74.06 g/m^2^). Baseline characteristics and echocardiographic measurements of the included participants are provided in *Table* *1*.

**Table 1:** Clinical, echocardiographic and laboratory parameters of included patients across left ventricular mass/height^2^ quartiles

Measurements	All patients (*n* = 398)	LV mass/height^2^				*p*‐value^a^	F−/chi^2^‐ value^a^	*p* for linear trend
		1st Quartile (*n* = 99)	2nd Quartile (*n* = 100)	3rd Quartile (*n* = 100)	4th Quartile *n* = 99			
		♀ <46.58 g/m^2^, ♂ <56.69 g/m^2^	♀ ≥46.58 g/m^2^ – <55.34 g/m^2^, ♂ ≥56,69 g/m^2^ – <65.7 g/m^2^	♀ ≥55.34 g/m^2^ – <62.88 g/m^2^, ♂ ≥65.7 g/m^2^ – <77.15 g/m^2^	♀ ≥62.88 g/m^2^, ♂ ≥77.15 g/m^2^			
**Clinical parameters**								
Female sex, *n* (%)	200 (50)	50 (51)	50 (50)	50 (50)	50 (51)	1	<0.01	–
Age (years)	60.2 ± 0.7	**57.3 ± 1.7** ^$$$^	57.2 ± 1.4^$$$^	60.8 ± 1.5^$^	65.4 ± 1.3	**0.0001**	7.04	**0.0012**
Time since first diagnosis (months)	33 ± 2.6	36 ± 4.9	35 ± 6.4	30 ± 4.8	31 ± 2.6	0.833	0.29	0.399
BMI (kg/m^2^)	25.0 ± 0.2	**22.2 ± 0.3** ^###,***,$$$^	24.4 ± 0.4^*,$$$^	25.6 ± 0.4	27.2 ± 0.4	**<0.0001**	21.04	**<0.0001**
Systolic blood pressure (mmHg)	131 ± 3	122 ± 2	129 ± 2	141 ± 11	132 ± 2	0.12	1.9	**<0.0001**
Diastolic blood pressure (mmHg)	78.8 ± 0.6	77.2 ± 1.1	80.4 ± 1.0	79.4 ± 1.1	78.1 ± 1.3	0.19	1.56	0.7789
ECOG, *n* (%)						0.10	18.86	–
0	70 (18)	19 (19)	24 (24)	12 (12)	15 (15)
1	152 (38)	44 (44)	40 (40)	38 (38)	30 (30)
2	106 (27)	21 (21)	23 (23)	26 (26)	36 (36)
3	63 (16)	14 (14)	12 (12)	20 (20)	17 (17)
4	7 (2)	1 (1)	1 (1)	4 (4)	1 (1)
Karnofsky index (%)	78 ± 0.8	79 ± 1.4	81 ± 1.6	74 ± 1.8*	77 ± 1.5	**0.014**	3.58	0.052
4‐m gait speed (m/s)	1.16 ± 0.02	1.16 ± 0.04	1.27 ± 0.04^*,$^	1.11 ± 0.05	1.11 ± 0.05	**0.032**	2.97	0.7513
Maximum handgrip strength (Newton)	307 ± 6	306 ± 12	317 ± 12	309 ± 13	296 ± 12	0.686	0.46	0.4054
10‐step stair‐climbing power (W)	362 ± 12	**302 ± 17** ^##,$^	396 ± 27	369 ± 26	381 ± 27	**0.0346**	2.93	**0.0412**
**Cancer and anticancer therapy details, *n* (%)**								
Cancer stage III/IV	325 (82)	83 (84)	78 (78)	83 (83)	81 (82)	0.77	1.14	–
Solid cancer	258 (65)	64 (65)	65 (65)	66 (66)	63 (64)	0.99	0.126	–
Relapse	94 (24)	29 (29)	21 (21)	26 (26)	18 (18)	0.26	4.07	–
Anticancer therapy naive	73 (18)	18 (18)	22 (22)	14 (14)	19 (19)	0.55	2.10	–
First‐line therapy	151 (38)	35 (35)	30 (30)	39 (39)	47 (48)	0.078	6.82	–
Major cancer surgery	95 (24)	26 (27)	25 (25)	24 (25)	20 (21)	0.80	1.01	–
Radiation therapy	119 (30)	31 (31)	32 (32)	28 (28)	28 (28)	0.90	0.55	–
Chest radiation	41 (10)	5 (5)	13 (13)	12 (12)	11 (11)	0.18	4.92	–
**Side diseases, *n* (%)**								
Anaemia	273 (69)	65 (66)	65 (65)	72 (72)	71 (72)	0.58	1.98	–
Arterial hypertension	170 (43)	**26 (26)** ^*,$$$^	35 (35)^$$$^	43 (43)^$$$^	66 (67)	**<0.0001**	37.79	–
Hypercholesterolaemia	114 (29)	14 (14)^**,$$$^	26 (26)^$^	33 (33)	41 (41)	**0.0002**	20	–
Type 2 diabetes mellitus	47 (12)	6 (6)^*,$$^	7 (7)^*,$^	16 (16)	18 (18)	**0.0108**	10.98	–
Chronic kidney disease	43 (11)	7 (7)	9 (9)	12 (12)	15 (16)	0.26	4.01	–
Cachexia	101 (26)	**40 (40)** ^#,**,$$$^	26 (26)^$^	24 (24)^$^	11 (11)	**0.0001**	22.29	–
**Medications**								
ACE inhibitors/ARBs	105 (26)	**13 (13)** ^$$$^	21 (21)^$$$^	25 (25)^$$$^	**46 (47)**	**<0.0001**	29.85	–
Beta‐blockers	74 (19)	**7 (7)** ^#,*,$$$^	19 (19)	19 (19)	29 (29)	**0.0006**	17.04	–
Anticoagulation	22 (6)	5 (5)	3 (3)	7 (7)	7 (7)	0.55	2.16	–
Diuretics	62 (16)	7 (7)^$$$^	13 (13)^$$$^	12 (12)^$$$^	**30 (30)**	**0.0001**	21.12	–
Antidepressants	47 (12)	12 (12)	8 (8)	19 (19)	8 (8)	0.0663	7.11	–
Opioids	79 (20)	18 (18)	14 (14)	27 (27)	20 (20)	0.14	5.41	–
Corticosteroids	123 (32)	27 (27)	35 (35)	32 (32)	32 (32)	0.7	1.44	–
**Echocardiography parameters**								
LV mass Dev. (g)	183 ± 3	133 ± 3	173 ± 3	195 ± 4	230 ± 5			–
LV mass/height^2^ (g/m^2^)	61.4 ± 0.7	44.7 ± 0.8	56.4 ± 0.6	65.1 ± 0.7	79.5 ± 1.0			–
LV mass/BSA (g/m^2^)	97.5 ± 1.1	74.4 ± 1.3	90.8 ± 1.2	102.7 ± 1.3	122.1 ± 1.9			–
LVEF (%)	64.3 ± 0.2	63.7 ± 0.4^##^	65.6 ± 0.4^**,$^	63.8 ± 0.4	64.1 ± 0.4	**0.0034**	4.63	0.3393
LV GLS (%)	−19.1 ± 0.2	−19.0 ± 0.4	−19.4 ± 0.3	−19.3 ± 0.3	−18.6 ± 0.4	0.43	0.92	0.4685
SV (ml)	54.8 ± 0.7	**50.0 ± 1.2** ^###,***,$$$^	55.2 ± 1.3	57.8 ± 1.4	57.4 ± 1.2	**<0.0001**	10.5	**<0.0001**
Heart rate (bpm)	76.1 ± 0.7	**78.6 ± 1.6** ^$$^	77.0 ± 1.5	75.6 ± 1.4	73.1 ± 1.2	**0.0457**	2.7	**0.0123**
Cardiac output (ml/min)	4110 ± 55	3848 ± 110**	4134 ± 118	4309 ± 106	4149 ± 106	**0.0278**	3.01	**<0.0001**
Cardiac output/height^2^ (ml/min/m^2^)	1395 ± 19	**1303 ± 37** ^**,$$^	1367 ± 39	1453 ± 35	1456 ± 39	**0.0091**	3.9	**<0.0001**
Cardiac index (ml/min/m^2^)	2219 ± 29	2166 ± 57	2192 ± 63	2286 ± 56	2232 ± 58	0.5	0.79	0.2068
LVEDV (ml)	89.1 ± 1.3	**78.0 ± 2.0** ^###,***,$$$^	92.1 ± 2.8	96.4 ± 2.7	90.0 ± 2.5	**<0.0001**	9.62	**<0.0001**
IVSd (mm)	10.7 ± 0.1	**9.44 ± 0.16** ^###,***,$$$^	10.5 ± 0.2^*,$$$^	11.0 ± 0.1^$$$^	11.9 ± 0.2	**<0.0001**	41.74	**<0.0001**
LVIDd (mm)	45.0 ± 0.3	41.5 ± 0.5^###,***,$$$^	45.2 ± 0.5^$$^	46.0 ± 0.5^$^	47.4 ± 0.5	**<0.0001**	25.58	**<0.0001**
LVPWd (mm)	9.57 ± 0.07	**8.61 ± 0.12** ^###,***,$$$^	9.23 ± 0.10^***,$$$^	9.86 ± 0.11^$$$^	10.59 ± 0.14	**<0.0001**	50.91	**<0.0001**
RWT	0.43 ± 0.004	0.42 ± 0.01^$$^	0.41 ± 0.01^$$$^	0.43 ± 0.01	0.46 ± 0.01	**0.0032**	4.67	**<0.0001**
LA volume (ml)	43.4 ± 0.6	36.3 ± 1.1^###,***,$$$^	42.9 ± 1.1^*,$$^	46.7 ± 1.3	47.9 ± 1.3	**<0.0001**	18.79	**<0.0001**
LAVI (ml/m^2^)	23.3 ± 0.3	20.4 ± 0.6^##,***,$$$^	22.6 ± 0.5^**,$$$^	24.7 ± 0.6	25.4 ± 0.6	**<0.0001**	15.79	**<0.0001**
Mitral E/A ratio	1.02 ± 0.02	1.14 ± 0.04^#,**,$$$^	1.03 ± 0.03^$^	0.99 ± 0.03	**0.91 ± 0.03**	**0.0001**	7.63	**<0.0001**
Mitral E/A ratio <1.00, *n* (%)	208 (52)	39 (40)	51 (51)	52 (53)	**66 (72)**	**0.0001**	20.27	**<0.0001**
Mitral E/e′ mean	8.03 ± 0.13	7.90 ± 0.26	7.49 ± 0.27^*,$$^	8.30 ± 0.24	**8.46 ± 0.25**	**0.0343**	2.91	**0.0236**
Mitral E/e′ mean >8.00, *n* (%)	169 (43)	47 (48)	33 (34)	47 (50)	42 (43)	0.11	6.14	0.8864
TAPSE (mm)	24.7 ± 0.2	**23.5 ± 0.3** ^###,***,$^	25.6 ± 0.3$$	25.1 ± 0.3	24.4 ± 0.3	**<0.0001**	8.35	0.1139
RA volume (ml)	33.4 ± 0.6	29.5 ± 0.8^***,$$$^	32.4 ± 1.0^$$^	34.8 ± 1.0	37.2 ± 1.4	**<0.0001**	9.18	**<0.0001**
RAVI (ml/m^2^)	17.9 ± 0.3	16.6 ± 0.4^*,$$$^	17.0 ± 0.4^$$$^	18.3 ± 0.5	19.5 ± 0.6	**<0.0001**	7.28	**<0.0001**
PASP (mmHg)	28.8 ± 0.4	27.9 ± 0.8^$^	27.3 ± 0.5^*,$^	29.8 ± 0.9	**30.1 ± 0.8**	**0.0201**	3.31	**0.0041**
**Laboratory parameters**								
Haemoglobin (g/dl)	11.5 ± 0.1	11.7 ± 0.2	11.4 ± 0.2	11.3 ± 0.2	11.4 ± 0.2	0.63	0.58	0.2471
Leucocytes (/nl)	7.85 ± 0.30	8.20 ± 0.68	7.02 ± 0.43	8.16 ± 0.59	8.05 ± 0.62	0.43	0.92	0.5355
Thrombocytes (/nl)	260 ± 7	270 ± 12	248 ± 11	271 ± 15	250 ± 15	0.43	0.91	0.1729
Sodium (mmol/L)	138.7 ± 0.2	138.7 ± 0.4	138.9 ± 0.3	138.5 ± 0.4	138.4 ± 0.4	0.79	0.35	0.8905
Potassium (mmol/L)	3.92 ± 0.02	3.88 ± 0.05	3.92 ± 0.04	3.92 ± 0.04	3.96 ± 0.05	0.71	0.46	0.3253
(hs)Troponin T (ng/L)	11 (6–17)	9 (5–13)	9 (5–18)	12 (7–21)	11 (8–17)	0.91	0.18	**0.0054**
NT‐proBNP (ng/L)	216 (97–557)	179 (82–545)^$$^	199 (95–459)^$$^	211 (97–559)	**334 (125–838)**	**0.0108**	3.77	**0.0169**
Creatinine (mg/dl)	0.85 ± 0.02	0.82 ± 0.03	0.83 ± 0.03	0.84 ± 0.03	0.89 ± 0.03	0.41	0.97	0.3281
eGFR (ml/min/1.73 m^2^)	87.6 ± 1.2	90.8 ± 2.4^$$^	90.5 ± 2.3^$$^	87.7 ± 2.3^$^	**81.2 ± 2.2**	**0.0108**	3.77	**0.0013**
GOT (U/L)	40.3 ± 2.4	36.3 ± 3.5	44.2 ± 6.3	45.6 ± 5.0	35.0 ± 3.4	0.28	1.27	0.5988
CRP (mg/L)	7.5 (2.2–25.0)	9.1 (2.4–34.4)	6.8 (2.1–23.0)	5.5 (2.2–25.4)	9.0 (2.5–21.1)	0.77	0.38	0.6914
Triglycerides (mg/dl)	147 ± 4	**132 ± 6** ^*,$^	137 ± 8^*,$^	159 ± 9	160 ± 8	**0.0131**	3.63	**<0.0001**
hGH (ng/ml) (*n* = 247; 66 vs. 64 vs. 60 vs. 57)	1.81 ± 0.16	**2.78 ± 0.37** ^###,**,$$^	1.29 ± 0.19	1.65 ± 0.31	1.46 ± 0.32	**0.0022**	5.01	**<0.0001**
IGF‐1 (ng/ml) (*n* = 248; 64 vs. 66 vs. 61 vs. 57)	81.3 ± 2.8	82.5 ± 5.6	87.9 ± 5.6	79.8 ± 5.0	74.1 ± 5.8	0.36	1.07	0.2139
log IGF‐1/hGH ratio (*n* = 243; 63 vs. 64 vs. 60 vs. 56)	1.95 ± 0.04	1.67 ± 0.07^###,**,$$^	2.11 ± 0.09	1.99 ± 0.09	2.04 ± 0.10	**0.0009**	5.67	**0.0034**

Normally distributed variables are presented as mean ± standard error of the mean, non‐normally distributed variables as median (interquartile range), and categorial variables as *n* (%). Values of specific interest are marked blue.

ACE, angiotensin‐converting enzyme; ANOVA, analysis of variance; ARB, angiotensin II receptor blocker; BMI, body mass index; BSA, body surface area according to the DuBois formula; CRP, C‐reactive protein; ECOG, Eastern Cooperative Oncology Group; E/e′, early diastolic filling velocity (E) over mitral annulus early diastolic tissue velocity (e′); eGFR, estimated glomerular filtration rate; GLS, global longitudinal strain; GOT, glutamic‐oxaloacetic transaminase; hGH, human growth hormone; hs, high‐sensitivity; IGF‐1, insulin‐like growth factor‐1; IVSd, interventricular septal thickness at end‐diastole; LA, left atrial; LAVI, left atrial volume index; LV, left ventricular; LVEF, left ventricular ejection fraction; LVEDV, left ventricular end‐diastolic volume; LVIDd, left ventricular internal diameter at end‐diastole; LV mass Dev., left ventricular mass according to the Devereux formula; LVPWd, left ventricular posterior wall thickness at end‐diastole; NT‐proBNP, N‐terminal pro‐B‐type natriuretic peptide; PASP, pulmonary artery systolic pressure; RA, right atrial; RAVI, right atrial volume index; RWT, relative weight thickness; SV, stroke volume; TAPSE, tricuspid annular plane systolic excursion.

^a^ANOVA *p*‐value/F‐value used for metric variables and chi^2^‐test and ‐value used for nominal variables. For 4‐m gait speed (m/s): *n* = 83 vs. 76 vs. 72 vs. 79; for 10‐step stair‐climbing power (W): (*n* = 51 vs. 54 vs. 47 vs. 48). Fisher's post‐hoc test: vs. 2nd quartile: ^#^ *p* < 0.05; ^##^ *p* < 0.01; ^###^ *p* < 0.001; vs 3rd quartile: *p < 0.05; **p < 0.01; ***p < 0.001; vs 4th quartile: ^$^ *p* < 0.05; ^$$^ *p* < 0.01; ^$$$^ *p* < 0.001. *p*‐values < 0.05 are bold.

### Clinical characteristics stratified by left ventricular mass

Patients in the lowest LV mass quartile (1st quartile) had lower BMI, fewer comorbidities (such as hypertension and hyperlipidaemia), were younger, more frequently cachectic, less frequently took antihypertensives, and had reduced 10‐step stair‐climbing power. Cancer stage, presence of solid tumours, and cancer relapse frequency were comparable across all LV mass subgroups. Frequencies of anticancer therapy‐naïve status, first‐line treatment, cancer surgery, and chest‐area radiation therapy were also similar across subgroups (*Table* *1*).

### Echocardiographic measurements stratification by left ventricular mass

**Figure 1:**
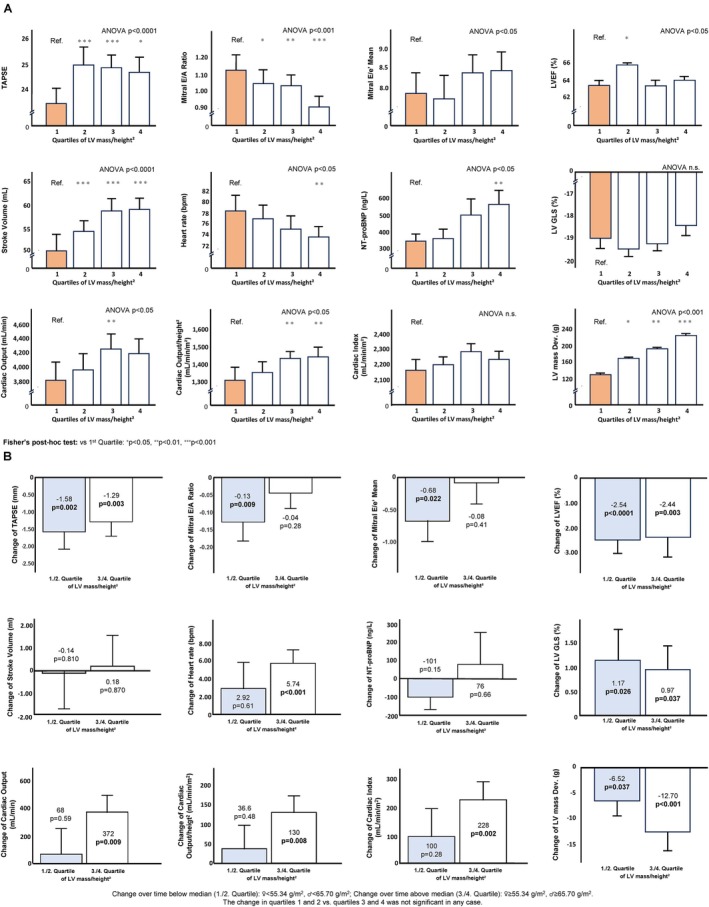
(*A*) Cardiology and echocardiographic characteristics of cancer patients according to quartiles of left ventricular (LV) mass/height^2^ (*n* = 398). (*B*) Changes of cardiology and echocardiographic characteristics of cancer patients during follow‐up according to lower/higher baseline LV mass/height^2^ (*n* = 143). GLS, global longitudinal strain; LVEF, left ventricular ejection fraction; NT‐proBNP, N‐terminal pro‐B‐type natriuretic peptide; TAPSE, tricuspid annular plane systolic excursion.

**Figure 2:**
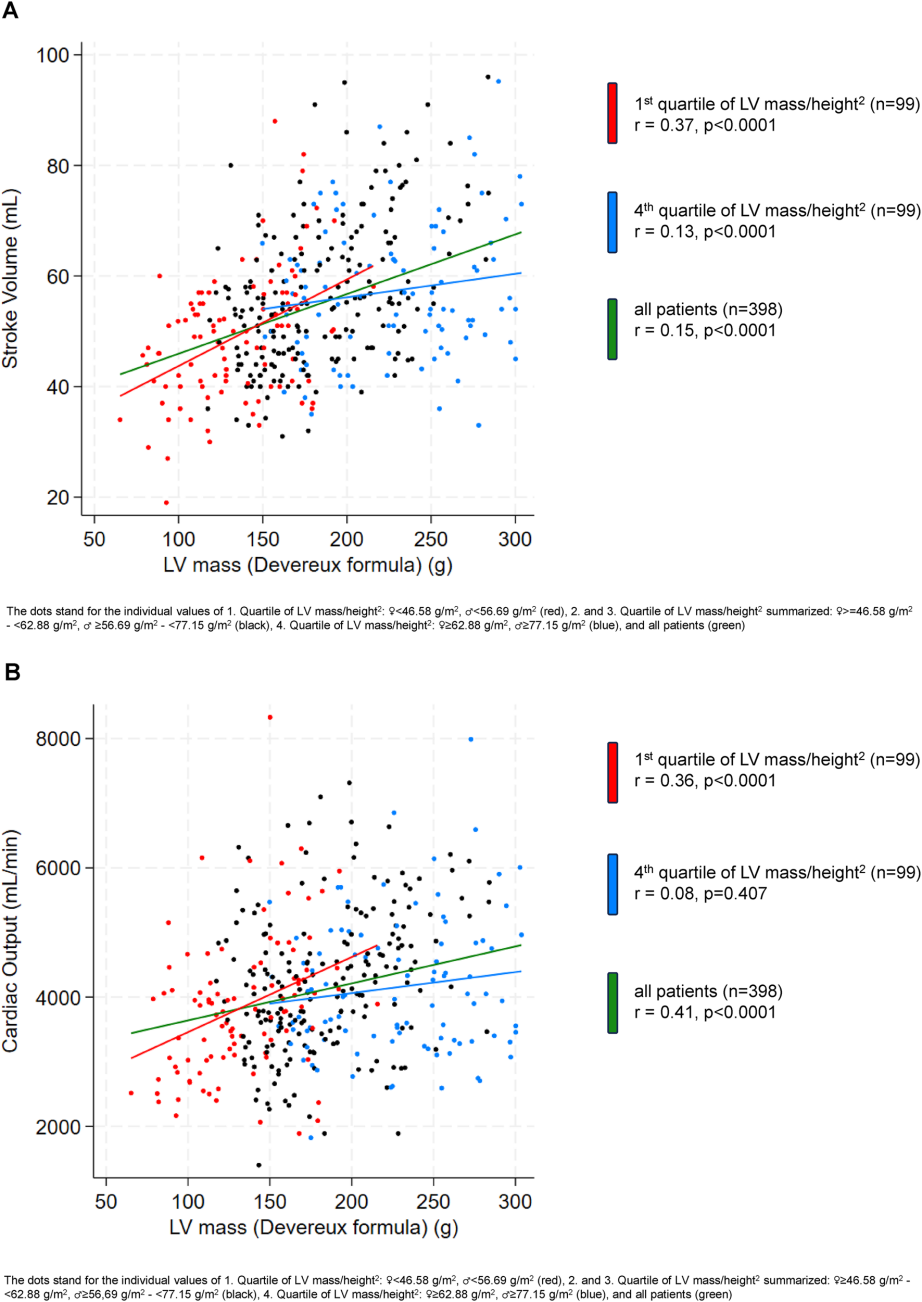
(*A*) Correlation of left ventricular (LV) mass with stroke volume according to 1st and 4th quartile of LV mass/height^2^ and in all patients (*n* = 398). (*B*) Correlation of LV mass with cardiac output according to 1st and 4th quartile of LV mass/height^2^ and in all patients (*n* = 398).

Left ventricular ejection fraction and global longitudinal strain (GLS) were normal across all quartiles – whereas in the in the 1st LV mass/height^2^ quartile, tricuspid annular plane systolic excursion (TAPSE), stroke volume, cardiac output, and cardiac output adjusted for height^2^ were lowest and heart rate was higher (*Figure* *1A*). LV mass correlation with stroke volume and cardiac output was particularly stronger towards the lower end of LV mass/height^2^ (*Figure* *2*). In the 4th LV mass/height^2^ quartile, mitral E/A was lowest and E/E′ was highest (*Figure* *1A*).

### Laboratory measurements stratification by left ventricular mass

Patients in the highest (4th) LV mass/height^2^ quartile had higher levels of N‐terminal pro‐B‐type natriuretic peptide (NT‐proBNP) levels and triglycerides. Troponin levels were similar among the different LV mass quartiles. Similarly, inflammatory markers such as C‐reactive protein were similar across LV mass quartiles. Glomerular filtration rate according to the Chronic Kidney Disease Epidemiology Collaboration formula was highest in the lowest LV mass quartile. In explorative analysis in 230 patients with available adrenaline and noradrenaline measurements, heart rate positively correlated with adrenaline and noradrenaline (*r* = 0.158, *p* < 0.0001; *r* = 0.171, *p* = 0.01) and stroke volume negatively correlated with adrenaline and noradrenaline (*r* = −0.134, *p* = 0.002; *r* = −0.130, *p* = 0.001).

### Follow‐up echocardiographic assessment

Overall, 143 patients had a follow‐up echocardiographic assessment, usually within 3–6 months. During the follow‐up period, patients below and above the median LV mass at baseline had a decrease in LV mass and a reduction in LV mass/height^2^ – which was more pronounced in patients above the median LV mass at baseline. However, the difference between the two groups was not statistically significant. These results remained consistent when LV mass was adjusted for height and BSA (*Figure* *1B*). LVEF decreased during follow‐up in patients with below and above the median LV mass – likewise, GLS worsened in both groups. The comparison of changes between the groups was not statistically significant (*Table* *2*, *Figure* *1B*).

**Table 2:** Repeat echocardiography of included patients divided in left ventricular mass/height^2^ below or above the median at baseline

	Change over time (below median) (*n* = 68)	Paired *t*‐test *p*‐value for change over time (below median)	Change over time (above median) (*n* = 75)	Paired *t*‐test *p*‐value for change over time (above median)	Unpaired *t*‐test *p*‐value (for change below vs. above median)	Change over time of all patients (*n* = 143)	Paired *t*‐test *p*‐value (change over time for all patients)
Change of BMI (kg/m^2^)	−0.84 ± 0.24	**0.006**	−0.53 ± 0.25	0.15	0.34	−0.68 ± 0.17	**0.004**
Change of LV mass Dev. (g)	−6.52 ± 3.12	**0.037**	−12.70 ± 3.70	**<0.001**	0.21	−9.77 ± 2.46	**<0.001**
Relative change of LV mass Dev. (%)	−2.54 ± 2.25	−5.59 ± 1.18	0.29	−4.14 ± 1.43
Change of LV mass/height^2^ (g/m^2^)	−1.95 ± 1.05	**0.047**	−4.46 ± 1.25	**<0.001**	0.14	−3.27 ± 0.83	**<0.001**
Change of LV mass/BSA (g/m^2^)	−1.54 ± 1.87	0.24	−6.12 ± 2.03	**0.003**	0.10	−3.94 ± 1.40	**0.003**
Change of LVEF (%)	−2.54 ± 0.53	**<0.001**	−2.44 ± 0.78	**0.003**	0.92	−2.49 ± 0.48	**<0.001**
Change of GLS (%)	1.17 ± 0.64	**0.026**	0.97 ± 0.49	**0.037**	0.81	1.07 ± 0.40	**0.002**
Change of SV (ml)	−0.14 ± 1.62	0.81	0.18 ± 1.40	0.87	0.88	0.04 ± 1.05	0.74
Change of cardiac output (ml/min)	68 ± 184	0.59	372 ± 121	**0.009**	0.17	238 ± 106	**0.024**
Change of cardiac index (ml/min/m^2^)	100 ± 97	0.28	228 ± 163	**0.002**	0.20	172 ± 56	**0.003**
Cardiac output/height^2^ (ml/min/m^2^)	37 ± 61	0.48	130 ± 42	**0.008**	0.21	89 ± 36	**0.016**
Change of heart rate in Echo (bpm)	2.92 ± 2.93	0.61	5.74 ± 1.54	**<0.001**	0.40	4.42 ± 1.59	**0.011**
Change of LVEDV (ml)	−1.23 ± 2.95	0.82	−4.46 ± 3.32	0.20	0.47	−2.93 ± 2.23	0.21
Change of IVSd (mm)	−0.25 ± 0.13	**0.020**	−0.28 ± 0.12	**0.005**	0.89	−0.27 ± 0.09	**<0.001**
Change of LVIDd (mm)	−0.37 ± 0.55	0.52	−1.01 ± 0.47	**0.044**	0.37	−0.70 ± 0.36	0.056
Change of LVPWd (mm)	−0.08 ± 0.11	0.47	−0.16 ± 0.13	0.44	0.64	−0.13 ± 0.09	0.34
Change of RWT	0.01 ± 0.01	0.27	0.01 ± 0.01	0.26	1.0	0.01 ± 0.01	0.11
Change of LV length (mm)	−2.06 ± 0.72	**0.004**	−1.81 ± 0.63	**<0.001**	0.79	−1.93 ± 0.47	**<0.001**
Change of LV width (mm)	−0.97 ± 0.62	0.10	−0.81 ± 0.74	0.54	0.87	−0.89 ± 0.49	**0.11**
Change of LA volume (ml)	−3.52 ± 1.23	**0.008**	−3.53 ± 1.27	**0.010**	0.99	−3.53 ± 0.88	**<0.001**
Change of LAVI (ml/m^2^)	−1.61 ± 0.68	**0.020**	−1.64 ± 0.72	**0.027**	0.98	−1.63 ± 0.49	**0.001**
Change of mitral E/A ratio	−0.13 ± 0.06	**0.009**	−0.04 ± 0.04	0.28	0.24	−0.08 ± 0.04	**0.007**
Change of mitral E/e′ mean	−0.68 ± 0.32	**0.022**	−0.08 ± 0.33	0.41	0.19	−0.36 ± 0.23	**0.032**
Change of TAPSE (mm)	−1.58 ± 0.50	**0.002**	−1.29 ± 0.42	**0.003**	0.65	−1.43 ± 0.32	**<0.001**
Change of RA volume (ml)	−1.15 ± 1.01	0.21	−1.15 ± 1.07	**0.021**	0.61	−1.65 ± 0.91	**0.011**
Change of RAVI (ml/m^2^)	−1.14 ± 0.70	0.16	−1.74 ± 0.83	**0.013**	0.58	−1.45 ± 0.55	**0.005**
Change of tricuspid valve velocity (m/s)	−0.01 ± 0.05	0.97	0.001 ± 0.045	0.67	0.90	−0.01 ± 0.03	0.69
Change of PASP (mmHg)	−0.02 ± 0.90	0.89	−0.27 ± 0.95	0.31	0.85	−0.15 ± 0.66	0.49
Change of NT‐proBNP (ng/L)	−101.13 ± 68	0.15	76 ± 175	0.66	0.54	−10.54 ± 95	0.91

BMI, body mass index; BSA, body surface area according to the DuBois formula; E/e′, early diastolic filling velocity (E) over mitral annulus early diastolic tissue velocity (e′); GLS, global longitudinal strain; IVSd, interventricular septal thickness at end‐diastole; LA, left atrial; LAVI, left atrial volume index; LV, left ventricular; LVEF, left ventricular ejection fraction; LVEDV, left ventricular end‐diastolic volume; LVIDd, left ventricular internal diameter at end‐diastole; LV mass Dev., left ventricular mass according to the Devereux formula; LVPWd, left ventricular posterior wall thickness at end‐diastole; NT‐proBNP, N‐terminal pro‐B‐type natriuretic peptide; PASP, pulmonary artery systolic pressure; RA, right atrial; RAVI, right atrial volume index; RWT, relative weight thickness; SV, stroke volume; TAPSE, tricuspid annular plane systolic excursion.

LV mass/height^2^ median ♀ = 55.34 g/m^2^, LV mass/height^2^ median ♂ = 65.70 g/m^2^, time between baseline and follow up = 140 ± 8 days (mean ± standard error of the mean).

Cardiac output increased overall and in patients above the median LV mass, while patients below the median had no change. Cardiac index increased overall and in patients above the median, whereas the change in patients below the median was not statistically significant. Similarly, heart rate showed an increase overall and in patients above the median LV mass, contrasting with no change in patients below the median. For TAPSE a decrease was observed overall and in patients below the median, and no change in patients above the median (*Figure* *1B*). Likewise, mitral E/A ratio showed a decrease overall and in patients below the median, while patients above the median demonstrated no change (*Table* *2*).

## Discussion

In this study assessing the clinical, echocardiographic and laboratory parameters related with cardiac wasting‐associated cardiomyopathy in advanced stage cancer patients, we report several key findings. First, patients with lower LV mass have a distinct clinical and echocardiographic phenotype characterized by higher heart rate, lower cardiac chamber volume, lower stroke volume, and reduced cardiac output, while presenting similar cardiac index and LVEF (*Graphical Abstract*). This pattern might be interpreted as a new form of heart failure, primarily driven by sympathetic activation. Second, patients with higher LV mass show another distinct phenotype with lower mitral E/A, higher E/E′ mean and higher NT‐proBNP levels, possibly representing beginning dilated cardiomyopathy with diastolic dysfunction. These findings suggest that several mechanisms contribute to cardiac wasting and the underlying pathophysiologic spectrum of cardiac wasting‐associated cardiomyopathy needs to be addressed with differential therapeutic strategies.

Patients in the lowest quartile of LV mass displayed distinct clinical characteristics, including younger age, lower BMI, having fewer comorbidities, more frequently showing signs of cachexia, and lower levels of triglycerides. These findings emphasize the clinical significance of the reduction in LV mass as an indicator of disease severity and its correlation with patient characteristics and general cachexia. Patients with the lowest LV mass demonstrated the lowest power on the 10‐step stair‐climbing test, possibly indicating a potential link between reduced LV mass and diminished functional capacity. These findings highlight the diverse effects of cardiac wasting on both heart function and total physical performance, emphasizing the importance of a comprehensive management strategy.

Patients in the lowest LV mass quartile also had the lowest cardiac chamber volumes and demonstrated higher heart rates compared with patients in the higher LV mass quartiles. The elevated heart rates could potentially be a compensatory response to the observed decrease in stroke volume^16^, ^17^ and are driven by sympathetic activation. The positive correlation between adrenaline/noradrenaline levels and heart rate and the negative correlation with stroke volume indicate that sympathetic overdrive has a deleterious effect on cardiac contractility and output, aggravating cardiac dysfunction. However, these patterns could also be influenced by reduced physical activity and deconditioning associated with prolonged hospital stays in cancer patients. Prior studies have demonstrated that even bed rest alone can lead to significant muscle atrophy, possibly further compounding the effects of cachexia and cardiac wasting.^18^ The relative contributions of cachexia versus deconditioning to LV mass reduction and reduced functional capacity warrant further investigation. These observed patterns correspond to the concept of cardiac wasting‐associated cardiomyopathy,^1^ which resembles a syndrome similar to heart failure in advanced cancer patients. The diminished amount of blood pumped per heartbeat, along with the compensatory rise in heart rate, indicates a dynamic interaction between the decrease in LV mass and cardiac physiological effort to sustain the amount of blood pumped by the heart.^16^, ^19^

Prior studies in other patient cohorts with anorexia nervosa or heart failure have also shown that a decreased LV mass is linked to LV wall thinning, LV size reduction, and LV stroke volume reduction.^20^, ^21^ Our study in cancer patients adds to these findings by exploring the clinical and detailed echocardiographic characteristics associated with varying degrees of cardiac wasting by stratifying cancer patients based on LV mass quartiles. As indicated by the filling time and lower volumes, the higher E/A ratio was possibly influenced by the shorter relaxation time. The worsening of LVEF, GLS, and TAPSE alongside loss of LV mass during follow‐up in all cancer patients (above and below the median LV mass) represents structural remodelling related to cardiac wasting. Despite these changes, cancer patients with baseline LV mass above the median where able to increase their cardiac output and cardiac index during follow‐up, whereas patients below the median where not able to. The changes in cardiac output were mainly driven by an increase of resting heart rates while stroke volumes remained unchanged.

The differences in human growth hormone, triglyceride, NT‐proBNP levels, across LV mass quartiles provide further insights into the interplay between biomarkers and cardiac wasting. Cancer patients with the lowest LV mass showed an increase in human growth hormone levels; a possible attempt of the body to counteract cardiac wasting.^22^ Alterations in lipid metabolism have also previously been implicated in the pathophysiology of wasting in patients with anorexia nervosa or cancer cachexia.^23^, ^24^, ^25^ Changes in triglyceride levels observed in our study emphasize the systemic impact of cardiac wasting with depletion of fat storages. Patients in the highest LV mass quartile had higher levels of NT‐proBNP, indicating that not only patients with low LV mass show cardiac dysfunction, but also patients with higher LV mass are experiencing increased myocardial stress and reduced diastolic dysfunction as indicated by higher E/E′ mean values, possibly representing beginning dilated cardiomyopathy.

This study confirms that patients with cardiac wasting‐associated cardiomyopathy exhibit a unique clinical presentation, which includes symptoms that are similar to those of heart failure, as well as adaptive changes such as lower stroke volume, higher heart rates, lower blood pressure, and more frequent anaemia.^26^ It remains uncertain whether cardiac wasting‐associated cardiomyopathy is a distinct entity that contributes to poor outcomes or a further presentation of cachexia that involves both cardiac and skeletal muscle wasting. Future investigations should focus on the impact of aberrant haemodynamics, which are frequently seen in the later stages of cancer, as well as the identification of the molecular mediators of cardiac wasting. Additionally, further studies are needed to distinguish cardiac wasting‐associated cardiomyopathy as a unique entity and not a manifestation of generalized cachexia compounded by deconditioning due to reduced physical activity during advanced cancer. While both involve changes in body composition, cardiac wasting is more closely linked to heart function and size, with cardiac wasting‐associated cardiomyopathy leading to altered metabolism, reduced cardiac output, and possibly fluid retention.

Our findings have important clinical implications, particularly in the context of cardio‐oncology trials, where cardiac wasting‐associated cardiomyopathy could be considered as a novel endpoint. Lena *et al*.^6^ demonstrated that a decrease in LV mass of 10% or more over a period of 3 to 12 months is clinically significant and is linked to impaired functional status and decreased overall survival. These proposed cut‐off values for LV mass and LV mass/height^2^ provide a foundation for potential validation in future clinical trials. This aligns with Asnani's recommendation that trials in advanced cancer patients should consider the loss of LV mass as a potential endpoint.^27^

Limitations in this study should be noted. First, the sample size for the follow‐up group should be even larger in future studies to further account for potential variability in response to treatment or intervention. This will be important to draw additional conclusions regarding the long‐term effects of cachexia and cardiac wasting. Furthermore, although the inclusion criteria are defined comparatively broad to depict a real‐world cohort representative for general oncology wards, they encompass various characteristics, including cancer type as well as the type and duration of anticancer therapy, which may also contribute to the progression of cachexia and cardiac wasting. Future studies with longer and repeat follow‐ups are warranted to confirm findings from this study and to explore long‐term outcomes.

In conclusion, patients with advanced cancer with low LV mass have a distinct clinical and echocardiographic phenotype characterized by lower cardiac chamber volumes, stroke volume, and cardiac output with normal LVEF and GLS that may be the distinct feature of cardiac wasting‐associated cardiomyopathy.

### Funding

This study was partly funded by the German Centre for Cardiovascular Research through research support to Dr M.S. Anker.

**Conflict of interest**: D.P.M. reports speaker fees and honoraria from Servier, Amgen, Merck, Sanofi, MSD, AstraZeneca, Pierre Fabre, GSK, Seagen, G1, IKF GmbH, Onkowissen, COR2ED, Taiho, Takeda, Incyte, Cureteq, 21up, Medscape, Aptitude Health, Regeneron; funding (institution): Servier, Amgen. U.K. has served on advisory boards for Roche, Janssen‐Cilag, Celgene, Takeda, Bristol Myers Squibb, Gilead, Hexal, Pfizer, AstraZeneca, and Pentixapharm; has received clinical research support from Janssen‐Cilag, Novartis, Takeda, Bristol Myers Squibb, Roche, and Pfizer; and has received travel support from Roche, Bristol Myers Squibb, Gilead, Takeda, Janssen‐Cilag, and Celgene. L.B. has received honoraria from AbbVie, Amgen, Astellas, Bristol Myers Squibb, Celgene, Daiichi‐Sankyo, Gilead, Hexal, Janssen, Jazz Pharmaceuticals, Menarini, Novartis, Pfizer, Roche, Sanofi, and Seattle Genetics; and has received research support from Bayer and Jazz Pharmaceuticals. M.T. reports speaker fees and travel support from AstraZeneca, Edwards Lifesciences, Novartis, Bayer, Daiichi Sankyo; is supported by a grant from the Deutsche Krebshilfe (70115402) and by the Deutsche Forschungsgemeinschaft (GRK 2989/1 TP P1). A.A.M. reports speaker fees and/or advisory boards from Amgen, Berlin Chemie, Daiichi Sankyo, Edwards, Novartis, Sanofi; research funding from Daiichi Sankyo, Edwards, all outside the submitted work. T.R. has received honoraria, lecture fees, and grant support from Edwards Lifesciences, AstraZeneca, Bayer, Novartis, Berlin Chemie, Daiicho‐Sankyo, Boehringer Ingelheim, Novo Nordisk, Cardiac Dimensions, and Pfizer, all unrelated to this work. M.K. is supported by a Clinician Scientist Professorship Grant from the Else Kroener‐Fresenius‐Foundation; and has received personal fees and grant support from Daiichi‐Sankyo, Adrenomed, Sphingotec, and Vifor Pharma, all outside the submitted work, and is a part‐time employee of 4TEEN4 Pharmaceuticals. C.G.T. is supported by two grants from the Italian Ministry of Health (PNRR‐MAD‐2022‐12 376 632, PNRR‐MCNT2‐2023‐12 376 981), outside the submitted work and C.G.T. reports honoraria or consultation fees from VivaLyfe, Univers Formazione, Solaris, Summeet, AstraZeneca, Myocardial Solutions, Medtronic; funding from Amgen and MSD; and is listed as an inventor of two patents related to heart failure, outside the submitted work. U.W. is supported by a Clinical Fellowship Grant from the Berlin Institute of Health; and has received speaker fees and/or contributions to congresses from Abbott, AstraZeneca, Bayer, Berlin Chemie, Bristol Myers Squibb, GE Healthcare, Pfizer, Philips, and Servier, all outside the submitted work. S.v.H. has been a paid consultant for and/or received honoraria payments from AstraZeneca, Bayer, Boehringer Ingelheim, BRAHMS, Chugai, Grünenthal, Helsinn, Hexal, Novartis, Pharmacosmos, Respicardia, Roche, Servier, Sorin, and Vifor; also reports research support from Amgen, AstraZeneca, Boehringer Ingelheim, Innovative Medicines Initiative (IMI), and the German Center for Cardiovascular Research (DZHK). J.B. is consultant to Abbott, American Regent, Amgen, Applied Therapeutic, AskBio, Astellas, AstraZeneca, Bayer, Boehringer Ingelheim, Boston Scientific, Bristol Myers Squibb, Cardiac Dimension, Cardiocell, Cardior, CSL Bearing, CVRx, Cytokinetics, Daxor, Edwards, Element Science, Faraday, Foundry, G3P, Innolife, Impulse Dynamics, Imbria, Inventiva, Ionis, Levator, Lexicon, Lilly, LivaNova, Janssen, Medtronics, Merck, Occlutech, Owkin, Novartis, Novo Nordisk, Pfizer, Pharmacosmos, Pharmain, Prolaio, Pulnovo, Regeneron, Renibus, Roche, Salamandra, Salubris, Sanofi, SC Pharma, Secretome, Sequana, SQ Innovation, Tenex, Tricog, Ultromics, Vifor, and Zoll. U.L. has received research support from Abbott, Amgen, Bayer and Novartis. All other authors have nothing to disclose.

## Supporting information


**Appendix S1.** Supporting Information.
