# Supplementary material for: Clinical and echocardiographic phenotype of cardiac wasting in patients with advanced cancer
Source: Eur J Heart Fail. 2025 Aug 6;27(11):2130–40. doi: 10.1002/ejhf.3744 (PMC12765034; doi:10.1002/ejhf.3744)
Supplement: Supplementary file 1 — Appendix S1. Supporting Information. [file EJHF-27-2130-s001.docx]

**Supplement Table 1. Cancer entity groups (n=398)**.

| Cancer Diagnosis | Solid /hematological cancer | Number of all patients, n (%) |
| --- | --- | --- |
| Breast cancer | Solid | 52 (13) |
| Choroid melanoma | Solid | 40 (10) |
| Lung cancer | Solid | 39 (10) |
| Colorectal cancer | Solid | 31 (8) |
| Testicular cancer | Solid | 22 (6) |
| Head and neck cancer | Solid | 19 (5) |
| Pancreatic cancer | Solid | 11 (3) |
| Prostate cancer | Solid | 11 (3) |
| Urothelial cancer | Solid | 6 (2) |
| Uterus carcinoma | Solid | 4 (1) |
| Salivary gland cancer | Solid | 4 (1) |
| Upper gastrointestinal cancer | Solid | 4 (1) |
| Thyroid cancer | Solid | 3 (1) |
| Ovarian cancer | Solid | 3 (1) |
| Other | Solid | 12 (3) |
| Non-Hodgkin Lymphoma | Hematologic | 97 (24) |
| Hodgkin Lymphoma | Hematologic | 27 (7) |
| Multiple myeloma | Hematologic | 13 (3) |

Other includes liver and bile cancer (n=2), cervix carcinoma (n=2), anal cancer (n=2), skin melanoma (n=1), carcinoma of ethmoidal sinus (n=1), vulva carcinoma (n=1), osteosarcoma (n=1), germinoma (n=1), and adenoid cystic carcinoma (n=1).
